# Post-transplant patients with COVID-19 associated acute respiratory distress syndrome, a role for Tociluzumab: A case series

**DOI:** 10.1016/j.rmcr.2020.101319

**Published:** 2020-12-09

**Authors:** M. Ladna, F.L. Villanueva, P.B. Maharrey, J. Lascano

**Affiliations:** a Division of Internal Medicine, Department of Medicine, University of Florida, Gainesville, FL, United States; b Division of Pulmonary, Critical Care and Sleep Medicine, Department of Medicine, University of Florida, Gainesville, FL, United States

**Keywords:** COVID-19, Acute respiratory distress syndrome, Tocilizumab, Respiratory failure, Critical care, LIST, ARDS, acute respiratory distress syndrome, COVID-19, coronavirus disease of 2019, CT, computerized tomography, IL, interleukin, CAR-T cell: chimeric antigen receptor T-cell

## Abstract

COVID-19 is the disease caused by SARS-CoV-2 that portends both a relatively high mortality rate as well as high rate of intensive care admission amongst all age groups; however effective therapy remains poorly characterized. Post-transplant patients are especially high risk and underrepresented in the literature. In these patients, cytokine release may play a significant role in the development of acute respiratory distress syndrome, raising the hypothesis that interleukin-6 inhibitors such as tocilizumab may be of benefit. Here, we describe two high-risk post-transplant patients who were treated with single-dose tocilizumab after intubation for moderate acute respiratory distress syndrome secondary to confirmed COVID-19 infection. Both patients recovered rapidly and were successfully extubated and discharged from the hospital without need for supplemental oxygen shortly thereafter, and their clinical improvement correlated with response in interleukin-6 levels. Tocilizumab appears to hold promise for critically ill COVID-19 patients who require mechanical ventilation when given shortly after intubation.

## 1 Introduction

Severe acute respiratory syndrome coronavirus 2 (SARS-CoV-2) is a novel beta coronavirus which originated in Wuhan, China in 2019. This virus causes coronavirus disease of 2019 (COVID-19). SARS-CoV-2 has a high virulence, no vaccine, no antiviral treatment, and relatively high mortality of 1.8–3.4%. Disease severity of COVID-19 necessitating admission to intensive care units is estimated at 5–11.5% amongst all age groups [1]. The United States is at particularly high risk for SARS-CoV-2-related mortality due to high prevalence of risk factors for poor outcomes, which include age greater than 65, cardiovascular disease, cerebrovascular disease, hypertension, diabetes mellitus, history of tobacco use, and chronic obstructive pulmonary disease [[2], [3], [4]]. Transplant patients are likely at higher risk given immunosuppression and underrepresented in current literature.

Several treatments are being actively investigated including hydroxychloroquine and azithromycin combination therapy, lopinavir/ritonavir, and remdesivir. These agents have shown antiviral activity against SARS-CoV-2 in vitro [[5], [6], [7]] as well as promise in humans [8,9], though this data is limited. Utility of these agents is likely limited to early disease since they theoretically function as antiviral; late complications of COVID-19 include acute respiratory distress syndrome (ARDS), which may be a result of cytokine storm. Cytokine storm is triggered by release of interferon gamma by T-cells which leads to release of tumor necrosis factor alpha, IL-6, and IL-10 by macrophages which can go on to cause significant lung injury and ARDS, as well as distributive shock and multisystem organ failure [10]. Studies have already shown that lopinavir/ritonavir did not have significant benefit when used late in disease course for varying severities of COVID-19, including ARDS [11,12].

Mortality of late stage ARDS secondary to COVID-19 is high, with studies showing mortality from 48% up to 90% once intubated and placed on mechanical ventilation, which is significantly higher than mortality associated with intubation for other viral pneumonias which is around 22% [13,14]. The reason for this higher mortality could be related to cytokine storm as critically ill COVID-19 patients have cytokine profiles resembling macrophage activation syndrome and secondary hemophagocytic lymphohistiocytosis with significant elevations in IL-1B, IL-2, IL-6, IL-17, IL-8 and tumor necrosis factor [15], with similar serologic markers such as elevated ferritin, elevated liver enzymes, and coagulopathies [16]. Presence of cytokine storm is further supported by significant proportion of COVID-19 patients who are intubated and requiring vasopressors for distributive shock despite no evidence of bacterial superinfection [17]. Thus, an effective therapy is in dire need.

IL-6 inhibitors such as tocilizumab and siltuximab/sarilumab have shown benefit in treatment of cytokine storm in chimeric antigen receptor T-cell patients. These agents have also shown promise for treatment of ARDS in severely ill COVID-19 patients via suppression of cytokine storm [18]. We present two critically ill post-transplant patients, each requiring intubation and mechanical ventilation for severe COVID-19 disease who were treated with single-dose tocilizumab and experienced rapid subsequent improvement at a large tertiary referral center in Gainesville, Florida, United States.

## 2 Case 1

**Fig. 1:**
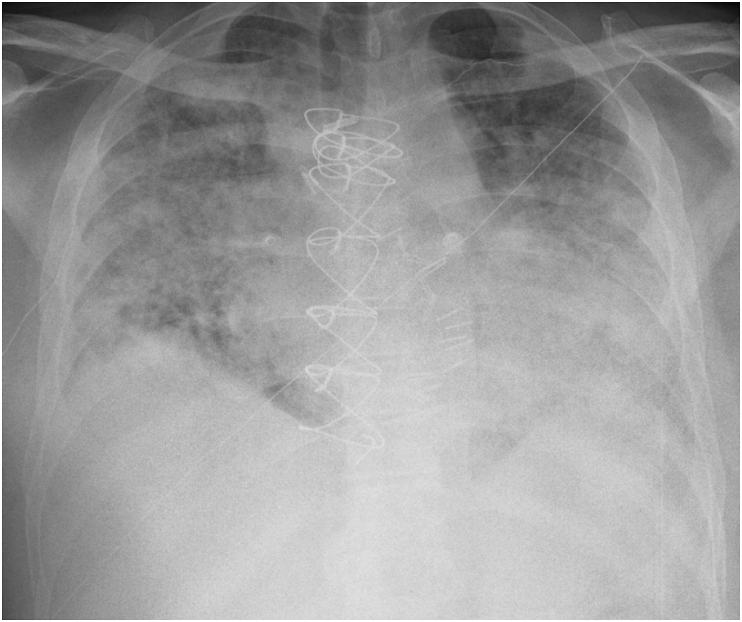
Chest radiograph of Case 1 on day of admission which shows bilateral multifocal airspace opacities.

**Fig. 2:**
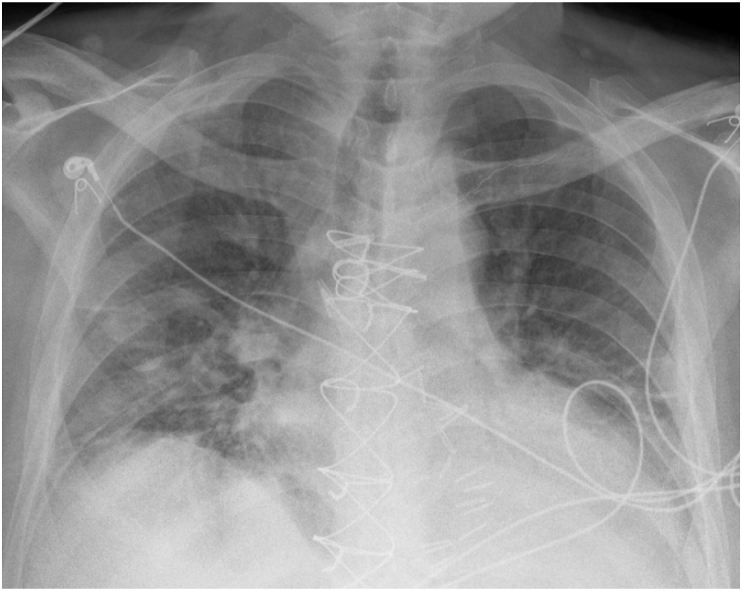
Chest radiograph of Case 1 on hospital day 8 post-extubation which shows improvement in multifocal airspace opacities.

Case 1 is a 50-year-old male with past medical history of coronary artery disease, nonischemic cardiomyopathy, type 2 diabetes mellitus, essential hypertension, and prior stroke who recently underwent kidney and heart transplant in February 2020. His post-transplant course was complicated with upper respiratory symptoms about one month after transplant, and his nasopharyngeal nucleic acid amplification test for SARS-CoV-2 returned positive. During that admission, he did not require supplemental oxygen, and was discharged home to complete a total five-day course of hydroxychloroquine. Three days after discharge, he presented to the emergency department for severe dyspnea. While being evaluated in the emergency department, he experienced rapid and profound hypoxic respiratory decompensation requiring intubation and mechanical ventilation. Initial lab work was significant for IL-6 level of 45 pg/mL, ferritin of 648 ng/mL, LDH of 426 U/L, D-Dimer of 4.84 UG/mL, high-sensitivity CRP of 74.9 mg/L. These elevated markers of inflammation and cell death were consistent with potential cytokine storm. Admission chest radiograph showed significant burden of bilateral airspace opacities (Fig. 1). He was given 400 mg of tocilizumab on his first hospital day, approximately 5 hours after intubation. He was also started on broad spectrum antibiotics (vancomycin and cefepime), azithromycin 500 mg daily, and hydroxychloroquine 200 mg twice a day. His transplant immunosuppression with tacrolimus was continued, he was started on stress dose hydrocortisone 50mg every 6 hours and his mycophenolate was held per International Society for Heart and Lung Transplantation guidelines for moderate-severe COVID-19 ARDS [19]. Due to progressively worsening hypoxia and ARDS (pAO_2_:FiO_2_ ratio of 117), he was started on inhaled epoprostenol and subsequently underwent brief neuromuscular blockade with cisatracurium for 4 h on his first hospital night. He continued to improve clinically in the subsequent days with supportive care and lung-protective ventilation per ARDSNet protocol [20], associated with rapid improvement in the airspace opacities seen on admission chest radiograph on repeat on his fourth hospital day (Fig. 2). He did not require proning or vasopressive support during his hospitalization. He was weaned off inhaled epoprostenol on his fifth hospital day, extubated on his seventh hospital day, and was discharged home without supplemental oxygen requirement on his eleventh hospital day. His IL-6 level peaked at 303 pg/mL and decreased to 21 pg/mL on the date of discharge.

## 3 Case 2

**Fig. 3:**
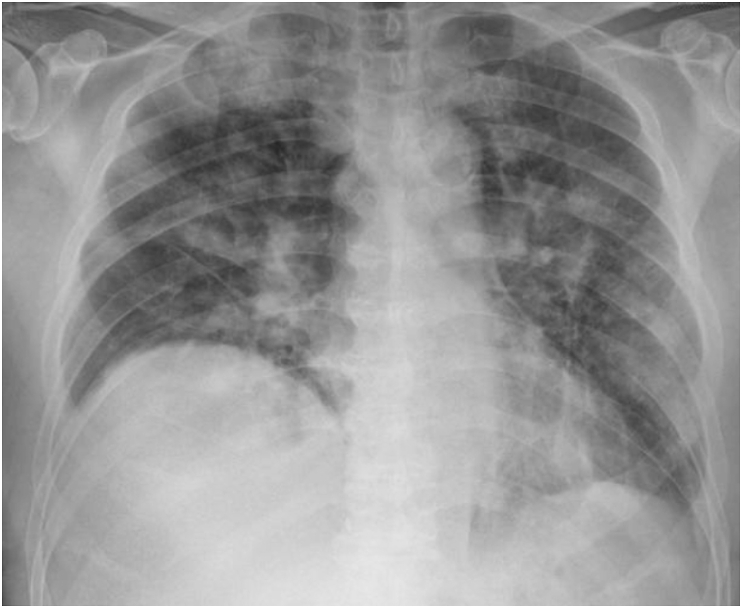
Chest radiograph of Case 2 on day of admission which shows bilateral multifocal airspace opacities.

**Fig. 4:**
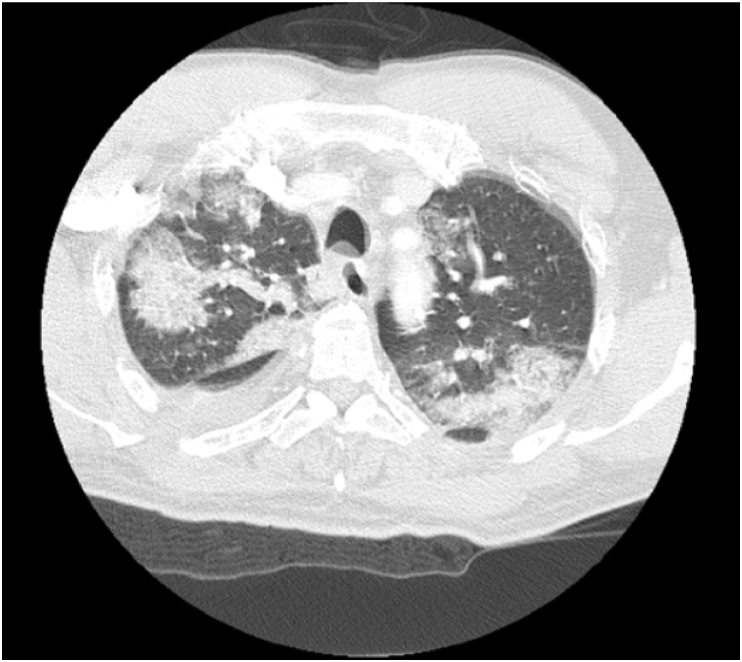
Axial CT chest on day of admission with multifocal mixed ground glass and dense consolidations throughout bilateral lungs.

**Fig. 5:**
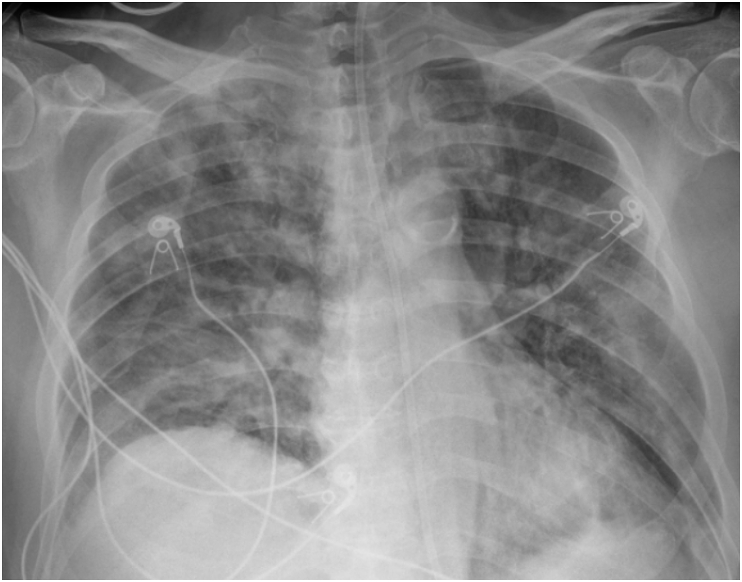
Chest radiograph of Case 2 on hospital day 4 with slight improvement in multifocal airspace opacities.

Case 2 is a 67-year-old male with past medical history of chronic hepatitis B complicated by hepatocellular carcinoma status post orthotopic liver transplant in September 2016, with repeat orthotopic Roux-en-Y liver transplant with pyloric exclusion in November of 2016, history of cryptococcus pneumonia with documented clearance after 28 days of fluconazole in 2018, hypertension, and type 2 diabetes mellitus who presented to the emergency department for one week of fever and dyspnea, and he was admitted to the medical intensive care unit due to acute hypoxic respiratory failure requiring intubation and mechanical ventilation. Admission chest radiograph showed multifocal airspace opacities (Fig. 3), and computerized tomography (CT) of the chest on day of admission showed multifocal mixed ground glass opacities and dense consolidations throughout bilateral lungs (Fig. 4). Initial arterial blood gas showed a pAO_2_:FiO_2_ ratio of 116, consistent with moderate ARDS. His nasopharyngeal nucleic acid amplification test for SARS-CoV-2 returned positive. His initial lab work was notable for IL-6 level of 31 pg/mL, ferritin of 320.4 ng/mL, LDH of 321 U/L, D-Dimer of 3.25 UG/mL, high-sensitivity CRP of 244 mg/L, which suggested a high level of inflammation and cell death consistent with potential cytokine storm. He was started on broad spectrum antibiotics (vancomycin and piperacillin/tazobactam), azithromycin 500 mg daily, and hydroxychloroquine 400 mg twice a day for one day followed by 200 mg twice a day. His home oral hydrocortisone for underlying adrenal insufficiency was continued and he did not require stress dose steroids. He was given single 400 mg dose of tocilizumab on his first hospital day, approximately 5 h after intubation, after which transplant hepatology recommended holding home mycophenolate mofetil and tacrolimus given potent immunosuppressive effects of tocilizumab. He was extubated on his second hospital day, and chest radiograph afterwards showed improvement in the multifocal airspace disease (Fig. 5). Tacrolimus was started seven days after administration of tocilizumab. He did not require neuromuscular blockade, vasopressive support, or proning during his hospitalization. He continued to improve and was discharged home on his eighth hospital day without supplemental oxygen requirement. His IL-6 level peaked at 318 pg/mL and decreased to 78 pg/mL on the date of discharge. His mycophenolate was restarted two weeks after administration of tocilizumab.

## 4 Discussion

We have presented two successful cases of single-dose tocilizumab use in critically ill post-transplant patients with moderate COVID-19 ARDS.

Although both patients had rapid improvement with tocilizumab, there are several unique factors. Notably, both patients were already immunosuppressed prior to hospitalization due to their post-transplant status, theoretically causing their respective immune systems to be less able to mount a strong cytokine release in the setting of COVID-19. This could perhaps explain how our cases differ from the results of the study by Lou et al. in Wuhan, China which showed that single-dose tocilizumab often failed to improve disease activity in those who were critically ill, who often needed multiple doses of tocilizumab due to persistently elevated IL-6 levels even when given in conjunction with high-dose glucocorticoids [18]. Both of our patients had a peak IL-6 level over 300 pg/mL during their hospitalization which subsequently trended downward prior to discharge. This particular population of post-transplant patients with severe COVID-19 is underrepresented in current literature and provides a unique perspective in use of novel therapies.

Of note, both patients were also treated with hydroxychloroquine and azithromycin since treatment took part prior to recent studies that revealed no benefit to COVID-19 patients when treated with hydroxychloroquine [21] with or without azithromycin [22]. In fact, some studies found an increase in adverse events for group treated with hydroxychloroquine [21]. Despite confounding factors and limitations, the rapid improvement seen in both patients is both promising and reassuring given that COVID-19 patients who require intubation typically require prolonged mechanical ventilation for average of 10 days [4]. Both patients also have several comorbidities, notably history of solid organ transplant on immunosuppression, and advanced age which puts them in higher risk for extended mechanical ventilation duration and mortality from COVID-19. The therapeutic window for IL-6 inhibitors such as tocilizumab is likely quite narrow. These agents must be given just before or immediately at onset of ARDS and cytokine storm, which tends to occur at day 7–10 of illness, and only in the minority of patients who experience rapid deterioration requiring admission to intensive care for mechanical ventilation (approximately 5–10% of all patients) [1]. Once ARDS has fully evolved, inhibition of immune system would likely do little to help remove proteinaceous fluid in alveoli, thus long mechanical ventilation and admission in the intensive care setting is to be expected.

Moving forward, further randomized controlled trials are necessary to support the findings we report. Given the robust response, we recommend consideration of tocilizumab, especially if other IL-6 inhibitors/blockers are not available, to critically ill patients with confirmed COVID-19 who require intubation for hypoxic respiratory failure with evidence of bilateral ground glass opacities on chest radiograph or CT, as aggressive empiric early treatment will likely have most impact on preventing ARDS and thus decreasing mortality.

## Funding sources

This research did not receive any grant support.

## Author contributions

ML, FLV, and PBM had full access to both cases in this study and drafted the original manuscript, and JL provided critical revision. ML, FLV, PBM, and JL were involved in the conception of the project and the approval of the final manuscript, and they will take responsibility for the integrity and accuracy of the data presented.

## Declaration of competing interest

The authors have no conflicts of interest to disclose.
